# Alcohol addiction, trichotillomania, and trauma: is there a secret link? A case report

**DOI:** 10.1192/j.eurpsy.2025.997

**Published:** 2025-08-26

**Authors:** M. Štracak, A. Boban, A. Krešo, M. Škarić, I. Todorić Laidlaw

**Affiliations:** 1 Psychiatry, “Ivo Pedišić” General Hospital, Sisak; 2 Psychiatry, “Dr. Ivan Barbot” Neuropsychiatry hospital, Popovača; 3 Psychiatry, “Vrapče” Universitiy Psychiatric hospital, Zagreb, Croatia

## Abstract

**Introduction:**

Interpersonal traumas affect women’s ability to rely on their social network to cope, which increases their reliance on maladaptive avoidance coping strategies such as drinking. According to psychodynamic models of trichotillomania hair pulling is a manifestation of unresolved sexual conflicts, disordered attachment, and dissociation from traumatic memories. (Houghton, D. C., Mathew, A. S., Twohig, M. P., Saunders, S. M., Franklin, M. E., Compton, S. N., Neal-Barnett, A. M., & Woods, D. W. (2016). Trauma and trichotillomania: A tenuous relationship. *Journal of Obsessive-Compulsive and Related Disorders*, *11*, 91-95.)

**Objectives:**

Evidence suggests that rather than making hair pulling worse, alcohol is often used to avoid negative affect related to hair pulling. It slows the nervous system’s activity and may also be used to reduce the urge to hair-pull. (Grant, J.E., Collins, M., Chesivoir, E. *et al.* Hazardous Alcohol Use in Trichotillomania. *Psychiatr Q* **94**, 361–369 (2023))

**Methods:**

We present a 43-year-old patient who has been hospitalized several times due to alcohol addiction. She is divorced, has no children, is unemployed, and finished elementary school. The patient reported that she started drinking at high school during weekend parties and gradually her drinking became more frequent. Now she is drinking a liter of vodka a day. Her last hospitalization was after she left a domestic violence shelter for women where she escaped because her partner abused her. After she was placed in the alcoholism ward, the initial psychic examination discovered alopecia. Childhood anamnesis showed sexual trauma at a young age, and she reported that she started to pull her eyelashes in kindergarten after two members of a family sexually abused her. Now she is wearing a wig and she reported that there are areas on her scalp where hair doesn’t grow anymore because she pulled it while she was in a shelter. As she described, pulling her hair gave her a sense of relief from anxiety.

**Results:**

During hospitalization, her pharmacotherapy was corrected and titrated. She was included in psychotherapy, family therapy, group, and individual psychotherapy. The therapeutic procedures applied have improved her mental condition. She has been advised to continue outpatient psychiatric treatment, to regularly use pharmacotherapy, and to attend family therapy and rehabilitation club.

**Conclusions:**

There is a clear need for future clinical studies not only in the area of trichotillomania’s relationship to substance use disorders but also in trauma coping mechanisms so we can truly understand how people adapt to and recover from trauma.

**Disclosure of Interest:**

None Declared

